# Cupid stealing visual attention - the restoration of Vermeer’s “Girl Reading a Letter at an Open Window” altered viewing behavior

**DOI:** 10.1177/20416695231205365

**Published:** 2023-11-14

**Authors:** Gregor U. Hayn-Leichsenring, Dana G. Rottleb

**Affiliations:** Institute for Anatomy I, University Hospital Jena, Jena, Germany

**Keywords:** aesthetics, art painting, eye tracking, johannes vermeer

## Abstract

A major restoration of Vermeer's “Girl Reading a Letter at an Open Window” revealed a painting of cupid on the back wall that had been overpainted. The uncovering of this painting within a painting changed the composition of the artwork. We performed an eye tracking study on digital representations of the painting to investigate how the restoration altered the way people perceive this artwork. We show that the painting of cupid draws visual attention from the letter and that viewing behavior depends on knowledge of the other version of the painting. Moreover, lay people prefer the version without cupid.

“Girl Reading a Letter at an Open Window” (ca. 1657) is a large oil painting by Johannes Vermeer (1632–1675). A major restoration conducted by the Gemäldegalerie Alte Meister in Dresden, Germany, revealed a painting of cupid on the back wall that had been overpainted with homogeneous paint after Vermeer's death (Koja et al., 2021). This had an impact on the paintings’ composition. To investigate the influence of the mentioned restoration on viewing behavior, we used two digital copies of the painting: The version as it was shown until 2017 (“wall version”) and the restored version as it is on display since 2021 (“cupid version”). Additionally to the exposed painting of cupid, a surface cleaning led to brighter and less yellowish colors in the cupid version.

A previous eye-tracking study comparing original art paintings with digitally altered versions of the same paintings showed an influence of object placement and brightness on viewing behavior (Quian Quiroga & Pedreira, 2011).

To conduct a detailed investigation, we defined five areas of interest (AOI: “letter”, “girl”, “wall”/“cupid”, “window” (including a reflection of the girl's face), “foreground”, Figure 1). 67 participants (49 female, 18 male, median age 21.0 years) participated in our study. In the first part (“Single paintings”), we asked participants to freely view both versions of the painting for 10 s (balanced study design). In the second part (“Side-by-side”), participants viewed both versions next to each other for 10 s (balanced study design) and were consecutively asked “Which version of the painting do you prefer?” and “Were you familiar with the paintings?”. Images were presented with a size of 248 × 323 mm (“Single paintings”) or 214 × 275 mm (“Side-by-side”) and a viewing distance of approximately 600 mm. For both parts, we monitored participants’ eye movements with a Tobii Pro X3–120 eye tracker. In our analysis, we focused on total fixation duration (TFD) for each AOI as a reliable measure for visual attention (Susac et al., 2019).

**Figure 1.**
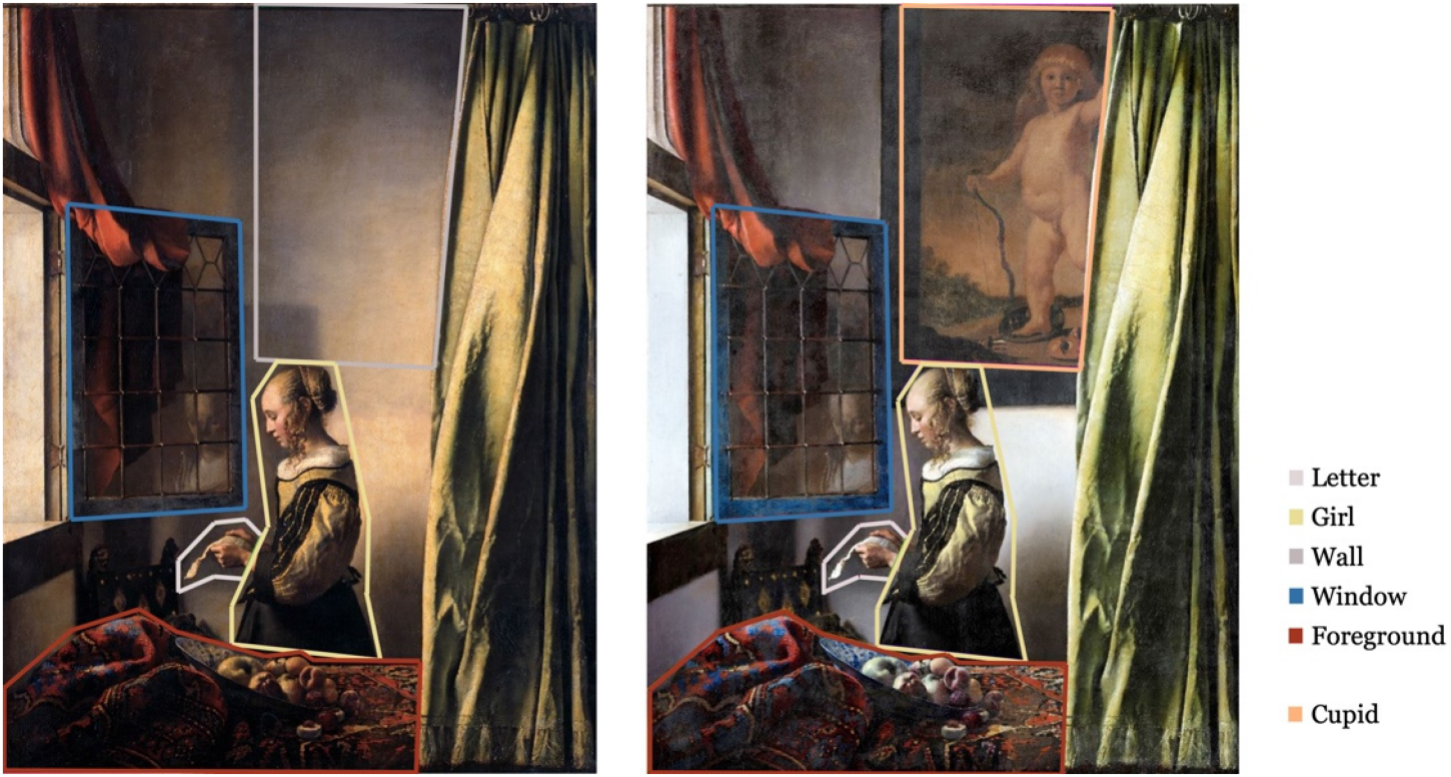
Display of AOIs.

Our results for part 1 (“Single paintings”) show that overall, in both versions longest TFD was on “girl” (Figure 2). Investigating the difference in viewing behavior between the versions, we analyzed data from participants who viewed the respective version first (Figure 2). The AOI “wall” in the wall version had an overall shorter TFD than “cupid” in the cupid version (independent samples student's t-test, *t*(65) = −7.377, *p* < .001). Furthermore, there was a longer TFD for “letter” (*t*(65) = 2.832, *p* < .01) in the wall version.

**Figure 2.**
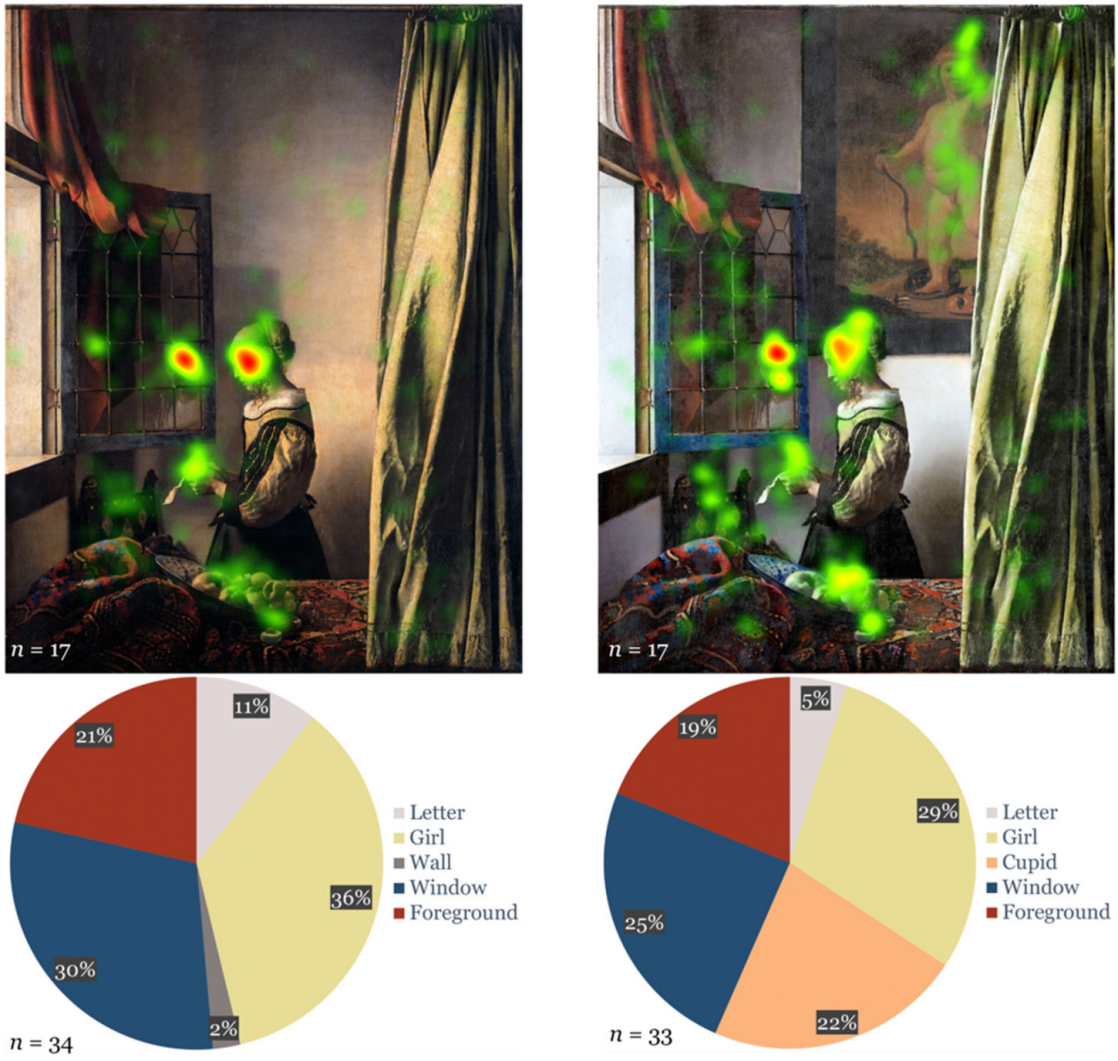
Heat map (half of participants) and diagram on TFD of AOIs in % for both versions when presented first.

Next, we investigated differences in viewing behavior depending on whether the respective version was presented before or after the other version. For the wall version when shown after the cupid version, we found longer TFD for “wall” (*t*(65) = 4.973, *p* < .001), and shorter TFD for “letter” (*t*(65) = −1.610, *p* < .01) and “foreground” (*t*(65) = 3.105, *p* < .01). For the cupid version when shown after the wall version, we found longer TFD for “cupid” (*t*(65) = 3.797, *p* < .001), and shorter TFD for “window” (*t*(65) = −3.139, *p* < .01) and “foreground” (*t*(65) = −2.294, *p* < .05) (Figure 3).

**Figure 3.**
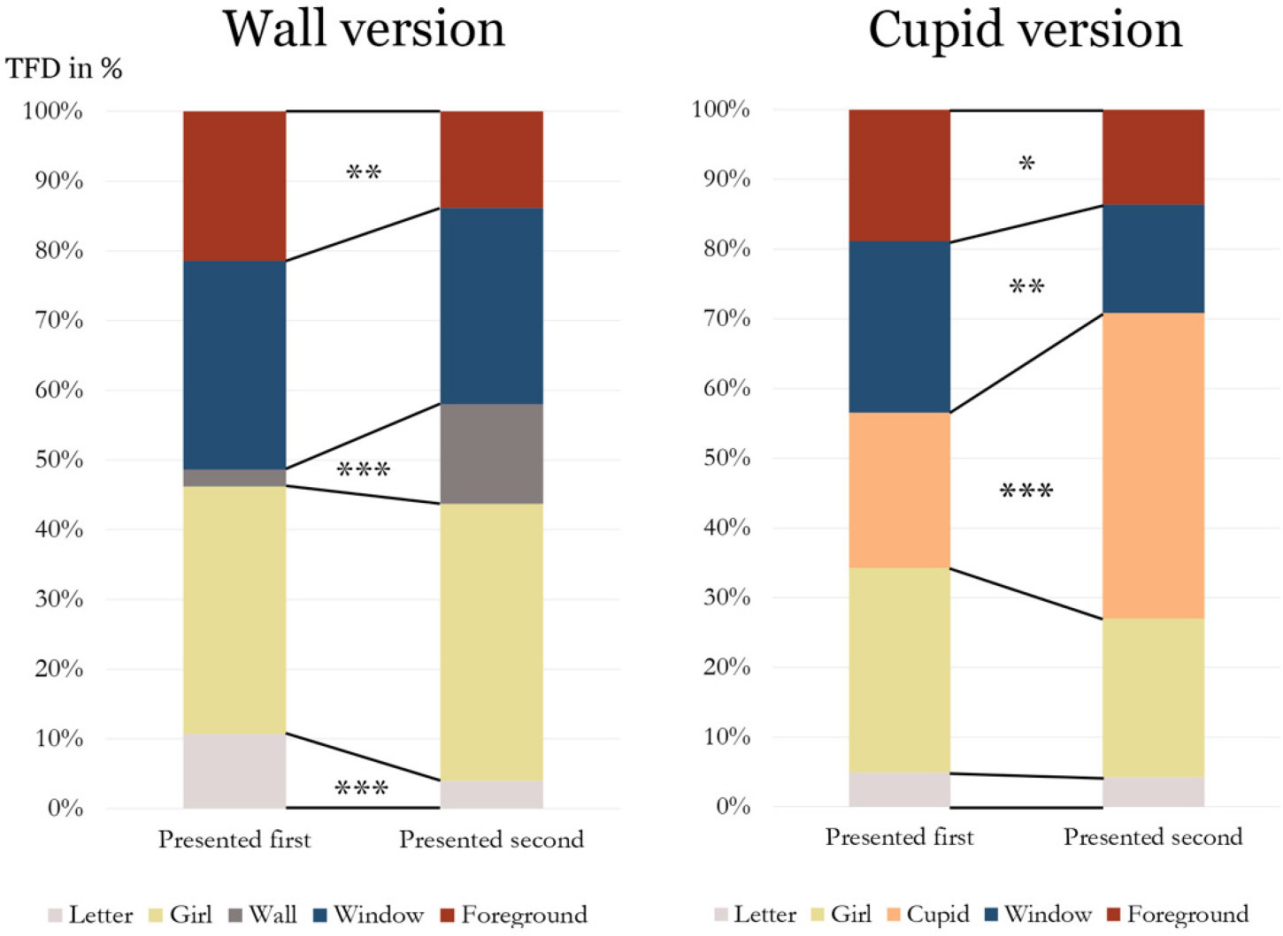
Differences of TFD for each AOI in percent depending on whether the respective version was presented first or second. ***p < .001; **p < .01; *p < .05. Sum of TFD: wall version: 5.508 s, cupid version: 5.222 s.

In part 2 (“Side-by-side”), fewer participants preferred the cupid version (15 *vs* 51; 1 undecided). Only 3 of 67 participants were familiar with the paintings before the experiment. Therefore, an analysis on familiarity was unfeasible.

Using the sum of TFDs from all AOIs of the respective version as a metric, we found overall shorter TFD for the wall version (*t*(66) = −2.002, *p* < .05; Figure 4). The effect was mainly driven by participants who preferred the cupid version; we found an interaction between preference and TFD with participants preferring the wall version looking relatively shorter at the cupid version (*t*(57) = −3.012, *p* < .01; Figure 4).

**Figure 4.**
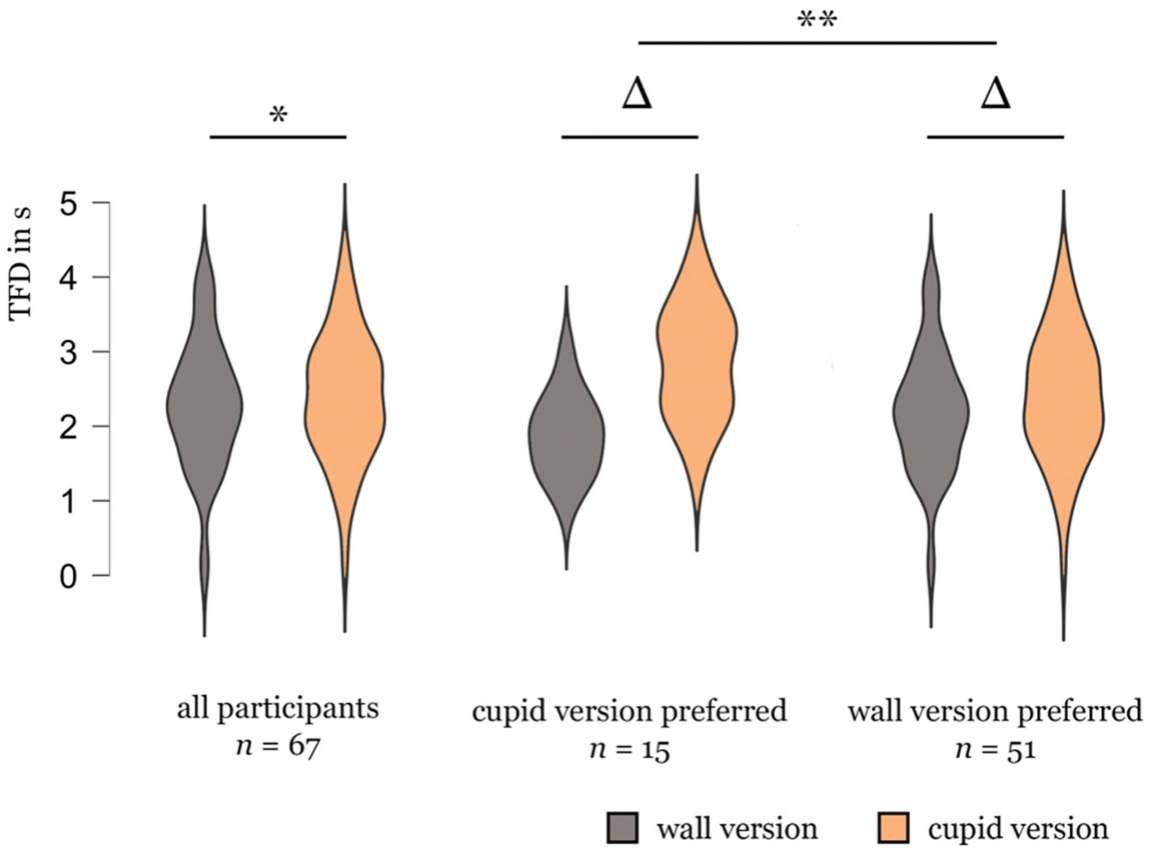
Violin plots on the interaction between preference and TFD. **p < .01; *p < .05.

While we attribute our findings to the revelation of the painting, we cannot rule out an effect of surface cleaning like it was demonstrated by Fontoura et al. (2023).

We showed that the painting of cupid attracts more visual attention than the blank wall, apparently drawing visual attention mainly from the letter, but not significantly from other elements in the painting. This finding is noteworthy, as scholars interpret the existence of the painting of cupid as a hint that the girl is holding a love letter (Koja et al., 2021) and, therefore, the depiction of cupid is considered to resolve the ambiguity of the letter. This could be the reason for the letter seeming to receive less visual attention in the cupid version as compared to the wall version. Furthermore, we found that consecutive viewing of the versions leads to shifting of visual attention towards the altered part of the painting (“wall”/“cupid”). Therefore, knowledge about the overpainted version might irretrievably change perceivers’ viewing behaviors. We speculate that this is true not only for our experiment, but also during encounters with the genuine artwork. Since it has been overpainted and the coverage has been removed, the painting of cupid probably receives more visual attention than it would have gotten if it had not been overpainted in the first place.

In our study, lay people overall preferred the wall version, but we refrain from making any judgment on the rationale to reveal the painting of cupid since implications of culture and originality cannot be measured with our method. However, we showed that the restoration of “Girl Reading a Letter at an Open Window” has an impact on viewing behavior.
